# Stress-related memories disrupt sociability and associated patterning of hippocampal activity: a role of hilar oxytocin receptor-positive interneurons

**DOI:** 10.1038/s41398-020-01091-y

**Published:** 2020-12-12

**Authors:** Mariah A. A. Meyer, Max Anstötz, Lynn Y. Ren, Michael P. Fiske, Anita L. Guedea, Viktoriya S. Grayson, Samantha L. Schroth, Ana Cicvaric, Katsuhiko Nishimori, Gianmaria Maccaferri, Jelena Radulovic

**Affiliations:** 1 Department of Psychiatry and Behavioral Sciences, Northwestern University, Feinberg School of Medicine, Chicago, IL 60611 USA; 2 Department of Physiology, Northwestern University, Feinberg School of Medicine, Chicago, IL 60611 USA; 3 Department of Obesity and Internal Inflammation, Fukushima Medical University, Fukushima, 960-1295 Japan; 4 Department of Neuroscience and Department of Psychiatry, Albert Einstein College of Medicine, The Bronx, NY 10461 USA

**Keywords:** Hippocampus, Molecular neuroscience

## Abstract

In susceptible individuals, memories of stressful experiences can give rise to debilitating socio-affective symptoms. This occurs even when the ability to retrieve such memories is limited, as seen in patients suffering from traumatic amnesia. We therefore hypothesized that the encoding, rather than retrieval, mechanisms of stress-related memories underlie their impact on social and emotional behavior. To test this hypothesis, we used combinations of stress-enhanced and state-dependent fear conditioning, which engage different encoding mechanisms for the formation of stress-related memories. We found that the encoding of stress-enhanced state-dependent memories robustly and sex specifically impairs sociability in male mice and disrupts the asymmetry of dentate gyrus (DG)/CA3 activity accompanying social interactions. These deficits were restored by chemogenetic inactivation of oxytocin receptor-positive interneurons localized in the hilus (Oxtr-HI), and by inactivation of dorsohippocampal efferents to the caudal lateral septum. Together, our data suggest that disrupted patterning of dorsohippocampal DG/CA3 activity underlies stress-induced sociability deficits, and that Oxtr-HI can be a cellular target for improving these deficits.

## Introduction

In susceptible individuals, autobiographical (episodic) memories of intensely stressful experiences can have debilitating consequences on social and emotional behavior and thus lead to social dysfunction, anxiety, or depression^1–3^. Interestingly, these symptoms are found both in patients suffering from post-traumatic stress disorder, who persistently retrieve such memories^4^, and in patients suffering from dissociative amnesia, whose access to such memories is partially or completely blocked^5,6^. Accordingly, abnormal functioning of episodic memory circuits, especially the hippocampus, has been found in both patient populations^7,8^, and additionally includes cortico-hippocampal inhibition in patients suffering from traumatic amnesia^8^.

Rodent models of stress-enhanced state-dependent fear conditioning (S-SDFC) and state-dependent fear conditioning (SDFC) allow studying the impact of stress-related memories on social behavior. Whereas increasing stress exposure strengthens the encoding of fear-inducing memory^9–13^, state-dependent encoding restricts memory access to specific brain states^14,15^. State-dependent memory is readily induced in mice trained on gaboxadol (GBX), an agonist of extrasynaptic GABA_A_R^16^ that enhances tonic inhibition in the DG^17^. Although such memories are normally not accessible to retrieval, their presence can be demonstrated when mice are tested in the presence of the drug^14,15^. Furthermore, in this model, stress-related fear memories seem to be inhibited by cortical mechanisms and preferentially processed by hippocampal–lateral septal (LS) circuits^14^.

Similar to humans, the rodent hippocampus plays a well-established role in the encoding of episodic-like memories^18,19^ and the regulation of social behaviors^20^, suggesting that approaches with rodents can provide mechanistic links on the interactions between memory and social behavioral systems. Mnemonic and social information is processed in the hippocampus through several parallel routes involving distinct subregions with unique functional contributions^21^. It is increasingly recognized that the processing of spatial and nonspatial components of episodic memories are segregated along the transverse axis of the hippocampus along the anatomically connected DG/CA3/CA1 subfields (i.e., suprapyramidal DG blade/proximodistal CA3/proximal CA1 and infrapyramidal DG blade/proximal CA3/distal CA1)^22–26^. It is not known, however, whether patterning of hippocampal activity contributes to the processing of social information and, if so, what are the underlying mechanisms.

Across a variety of brain regions, including the LS, nucleus accumbens, and prefrontal cortex, one general mechanism by which social behaviors are orchestrated are through the actions of the neuropeptide oxytocin and its receptor (Oxtr)^27–29^. While a growing number of studies have begun to shed light on Oxtr signaling in hippocampal CA3 and CA2 in social behavior^30^, Oxtr-positive neurons in the dentate gyrus (DG) have received little attention. By using mouse models of stress-enhanced and state-dependent memories, we set out to determine the neurobiological mechanisms underlying stress-induced social behavioral deficits, focusing on the patterning of DG/CA3 activity and the contribution of Oxtr-positive DG neurons. We found that the encoding of stress-related state-dependent memory disrupted social interactions and blunted the asymmetric patterning of DG/CA3 immediate early gene activity in response to social stimuli. Both effects could be restored by inhibition of Oxtr-positive DG hilar interneurons (Oxtr-HI), and by chemogenetic inactivation of hippocampal-LS projections. Together, these studies identify novel cellular and circuit mechanisms that contribute to adaptive and maladaptive, stress-mediated, responses to social stimuli.

## Materials and methods

### Mice

We used male and female wild-type C57BL/6J mice; and male Oxtr-reporter, Oxtr-Cre, and Oxtr^loxP/loxP^ mice. C57BL/6J mice were purchased from Harlan, Indianapolis, IN. Oxtr transgenic lines were obtained from K.N. and were backcrossed for six to nine generations with C57BL/6N mice. The Oxtr-reporter mice express Venus in neurons containing Oxtr, as described elsewhere^31^. The Oxtr-Cre mice^32^ express Cre in Oxtr-expressing neurons. Oxtr^loxP/loxP^ mice^33^ are a conditional knockout line that permit Cre-recombinase-dependent deletion of Oxtr. All litters were used for behavioral experiments and randomized by assigning similar numbers of littermates to different treatment conditions.

All animal procedures used in this study were approved by the Northwestern University IACUC and complied with federal regulations set forth by the National Institutes of Health.

### Contextual fear conditioning paradigms

**Fig. 1:**
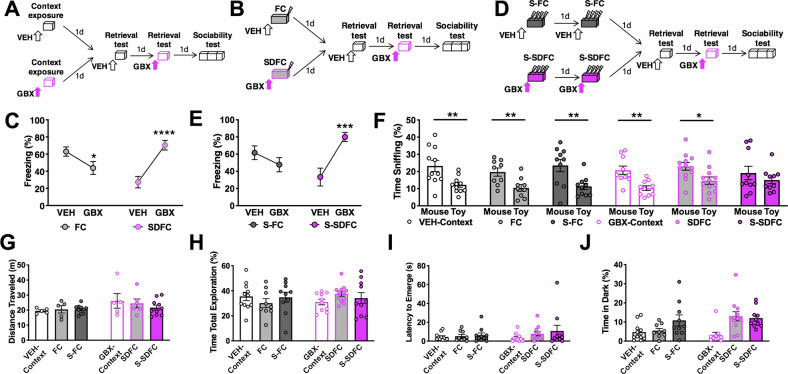
Effects of stress-related memory on sociability in males. **a** Schematic outline of the drug control experiment. Mice were implanted with cannula targeting the DG. Three days later, mice were injected i.h. with VEH or GBX before exposure to a context, in the absence of footshock (VEH-Context or GBX-context groups, respectively). On alternating days, mice were injected i.h. with VEH or GBX prior to reexposure to the context. Following, mice were tested for sociability. **b** Schematic outline of the behavioral paradigm. Mice were implanted with cannula targeting the DG. Three days later, mice were injected i.h. with VEH or GBX before a 1-day one-shock contextual fear conditioning procedure (FC or SDFC, respectively). On alternating days, mice were tested for memory retrieval on VEH or GBX by reexposure to the conditioning context without shock. Following memory retrieval tests, mice were subsequently tested for sociability. **c** During the memory retrieval tests, FC mice froze significantly less at the GBX test than at the VEH test. In contrast, SDFC mice froze significantly more in the presence of GBX than in the presence of VEH. *n* = 9FC/12SDFC mice per group; test drug *F*_(1,19)_ = 7.792, *P* = 0.0116; conditioning drug *F*_(1,19)_ = 0.3215, NS; interaction *F*_(1,19)_ = 52.67, *P* < 0.0001. **d** Schematic outline of the stress-enhanced behavioral paradigm. Mice were implanted with cannula targeting the DG. Three days later, mice were injected i.h. with VEH or GBX before a 2-day eight-shock contextual fear conditioning procedure (S-FC or S-SDFC groups, respectively). On alternating days, mice were tested for memory retrieval on VEH or GBX by reexposure to the conditioning context without shock. Following memory retrieval tests, mice were tested for sociability. **e** During the memory retrieval tests, S-FC mice froze similarly across both tests. S-SDFC mice froze significantly more in the presence of GBX than in the presence of VEH. *n* = 10S-FC/10S-SDFC mice per group; memory retrieval test drug *F*_(1,18)_ = 4.904, *P* = 0.0399; conditioning drug *F*_(1,18)_ = 0.4791, NS; interaction *F*_(1,18)_ = 16.74, *P* = 0.0007. **f** During sociability testing, VEH-Context, FC, S-FC, GBX-Context, and SDFC mice spent significantly more time with the mouse when compared with the toy. *n* = 11VEH-Context mice *t* = 3.119, df = 20, *P* = 0.0054; *n* = 9FC mice *t* = 2.98, df = 16, *P* = 0.0088; *n* = 10S-FC mice *t* = 3.038, df = 18, *P* = 0.0071; *n* = 10GBX-Context mice *t* = 3.772, df = 18*, P* = 0.0014; *n* = 12SDFC mice *t* = 2.698, df = 22, *P* = 0.0131. S-SDFC mice did not display a preference between mouse and toy. *n* = 10S-SDFC mice *t* = 0.9109, df = 18, *P* = 0.3744. **g** Activity during sociability was enhanced by GBX, but did not differ between individual groups. *n* = 5VEH-Context/5FC/10S-FC/5GBX-Context/6SDFC/10S-SDFC mice per group; stress *F*_(2,35)_ = 0.3696, NS; conditioning drug *F*_(1,56)_ = 4.249, *P* = 0.0468; interaction *F*_(2,35)_ = 0.7658, NS. **h** Total time spent sniffing both stimuli during sociability did not differ between groups. *n* = 11Veh-Context/9FC/10S-FC/10GBX-Context/12SDFC/10S-SDFC mice per group; stress *F*_(2,57)_ = 0.09279, NS; conditioning drug *F*_(1,57)_ = 0.02330, NS; interaction *F*_(2,57)_ = 1.294, NS. **i** Latency to emerge from dark box during light-dark emergence paradigm did not differ between groups. *n* = 11Veh-Context/9FC/10S-FC/10GBX-Context/12SDFC/10S-SDFC mice per group, stress *F*_(2,57)_ = 1.136, NS; conditioning drug *F*_(1,57)_ = 0.5803, NS; interaction *F*_(2,57)_ = 0.4048, NS. **j** Time spent in dark box was significantly increased by stress. *n* = 11Veh-Context/9FC/10S-FC/10GBX-Context/12SDFC/10S-SDFC mice per group, stress *F*_(2,56)_ = 7.51, *P* = 0.0013; conditioning drug *F*_(1,56)_ = 2.187, NS; interaction *F*_(2,56)_ = 2.733, NS. *P < 0.05; **P < 0.01; ***P < 0.001; ****P < 0.0001.

In most experiments, we used four versions of the contextual fear conditioning paradigm. Mice were exposed for 3 min to context, followed either by a single footshock (FC; Fig. 1b) or eight, pseudo-randomly delivered footshocks (S-FC; Fig. 1d) delivered over 2 days (3 s, 0.8 mA, constant current each). Two additional groups were identically trained, but on GBX to induce SDFC or S-SDFC (Fig. 1b, d). Only in one experiment, we increased the number of shocks to 32 (S-FC32, Fig. S1a). Fear conditioning was performed in an automated system (TSE Systems). Memory retrieval testing was always performed 24 h after the last conditioning trial and consisted of 3 min in the conditioning context. Freezing was scored using a sampling method (every 10 s by a trained observer) in parallel with automatic recording of activity using an infrared beam system^34^. Freezing was expressed as a percentage of the total number of observations during which the mice were motionless. All behavioral tests were performed by experimenters who were blind to drug and viral treatments.

### Sociability

Sociability was tested, as previously described^35^. Briefly, the chamber was a glass rectangular box divided into three compartments with a wire cylinder placed in each side compartment. The stimulus mouse was an age-, sex-, and background-matched non-littermate. Testing consisted of a 5 min habituation phase, in which no stimuli were present; followed by a 5 min sociability test, in which a stimulus mouse and an inanimate toy mouse were present in separate chambers. Animals’ position and motion were tracked by a top-mounted video camera connected to a computer and a DVD recorder and analyzed using Videomot II tracking software (TSE Systems). Videos were scored manually for time spent sniffing the cylinder (direct snout- or four-paw-to-cylinder contact) by an investigator blind to animal treatment identity. Sniffing time was converted to % of total test duration (5 min).

### Image analysis and quantification

For all analyses, flat images were obtained and equal light or laser intensity was applied to all captures. For analysis of Oxtr-positive cell populations, images were obtained using a ×5 objective on a Nikon Ti2 widefield microscope, with Nikon NIS-Elements software used for analysis. Counts were performed in the defined area of the hilus. For each given cell-type marker, using an automated script for counting, a set of training images was randomly selected and the marker-positive cells were manually annotated by an expert using a (a) background subtraction with a rolling ball radius of X μm, (b) signal diameter > X μm, and (c) intensity above Y (a.u). Proportions were expressed as the total number of Oxtr-positive or marker-positive neurons, respectively.

For TdTomato (TdT) and cFos analyses, images were obtained using a ×5 objective on an Olympus Fluoview FV10i confocal microscope using Lecia Application Suite software, and analyzed with ImageJ (NIH). Counts were normalized to blade area, DG area, and proximodistal CA3. Proximal frames were defined as the most proximal aspect of CA3 stratum pyramidale not enclosed by the DG blades. Distal frames were defined at the midline of the fimbria, as this landmark defines CA3a from CA3b^36^. For investigation of cFos in the caudal LS, a 700 × 300 µm^2^ counting frame was defined below the corpus callosum at a level defined +0.38 mm anterior from bregma. A constant threshold was used to distinguish cells as follows: signal diameter >5 μm and intensity above 0.47 (a.u.). All cell counts were performed using two sections per mouse, unless prohibited by lesion, and quantifications were completed by an experimenter blind to treatment condition.

To confirm inhibition by TetTox, analysis of vesicle-associated membrane protein 2 (VAMP2), which is essential for activity-dependent neurotransmitter release from presynaptic terminals^37^, was conducted. Images were obtained using an Olympus Fluoview FV10i confocal microscope. Pixel intensity scans were performed with the Just ImageJ analysis program. Pixel intensity was measured along a line ranging from the ventral border of the DG hilus to the dorsal border of the DG stratum moleculare. Pixel florescence intensities are indicated as relative units from 0 to 60.

Additional materials (antibodies, drugs, and viruses) and methods (genotyping, housing, behavioral tests, surgeries, infusions, immunohistochemistry, electrophysiological recordings, morphological reconstruction, etc.) are included in Supplementary information.

### Statistical analyses

Statistical analyses were performed using Graphpad. All samples were normally distributed, as determined with a one-sample Kolmogorov–Smirnov test. Homogeneity of variance was confirmed with Levene’s test for equality of variances. Grubb’s outlier test was used to determine outliers in all behavioral and cell count analyses, which were excluded. Across the behavioral studies, two outliers were identified. Two outliers were also identified from the cell count analyses. After the completion of behavioral testing, all brains were collected and cannula placements (Figs. S2 and S3) and virus spread were confirmed by immunohistochemical analysis of eGFP or mCherry signals. Two-tailed unpaired Student’s *t* test were used to determine within-group sociability preference related to sniffing mouse or toy, as recommended by Dr. Crawley who developed the paradigm that we used^38^. Between-group comparisons were performed by unpaired *t*-test, and one- or two-way ANOVA where appropriate. Significant *F* values were followed by post hoc comparisons using Bonferroni’s multiple comparison test. Correlation between the average eGFP and VAMP2 florescence intensities, and cFos analyses and sociability index was determined using Pearson’s correlation coefficient. Statistical differences were considered significant for all *P* values < 0.05. Group sizes were determined using power analyses assuming a moderate effect size of 0.5. All key findings were replicated at least twice.

All source data for the preparation of graphs and statistical analysis are presented online. All other relevant data that support the conclusions of the study are available from the authors upon request.

## Results

### Stress-enhanced state-dependent fear conditioning (S-SDFC) disrupts sociability in males

To determine the effects of stress-enhanced and state-dependent memories on social behavior, we tested the sociability of mice after context exposure, FC, or SDFC, respectively. In a control experiment for drug treatment, male mice were injected intrahippocampally (i.h.) with VEH or GBX 20 min before context exposure (VEH-Context and GBX-Context groups, respectively). One and 2 days later, mice were subjected to memory retrieval tests, one off-drug and one on-drug (VEH and GBX), to confirm memory formation. Freezing behavior was scored as a memory-induced index of fear^39,40^. Three days later, mice were subjected to a sociability test (Fig. 1a). In mice exposed to context, the analysis of freezing behavior during the VEH and GBX retrieval tests revealed no freezing (maximum average freezing 7%, data not shown). In a similar set of experiments but with stress elicited by a one-trial context-shock pairing (FC and SDFC groups, respectively) (Fig. 1b) analysis of freezing behavior during the VEH test revealed intact memory in FC mice and impaired memory in SDFC mice (Fig. 1c). In mice that were pre-injected i.h. with VEH (S-FC) or with GBX (S-SDFC) before eight context-shock pairings presented over 2 consecutive days (Fig. 1d), such that stress was enhanced, S-FC mice showed similar freezing at both VEH and GBX tests, demonstrating enhanced fear memory retrieval in response to more intense stress. In contrast, S-SDFC mice showed impaired memory retrieval when tested on VEH, but intact retrieval when tested on GBX, typical of state-dependence (Fig. 1e).

All of the groups exhibited a significant preference for the mouse when compared to the toy during social interaction, except for S-SDFC mice, which did not show social preference (Fig. 1f). A further increase of stress intensity to 32 pairings delivered over 4 consecutive days was similarly ineffective (Fig. S1a–d). These results suggest that the formation of state-dependent stress-related memories results in disrupted social behavior.

As compared to all other groups, S-SDFC did not involve changes of activity (Fig. 1g) or total stimuli exploration during the sociability test (Fig. 1h). These results indicate that the disruption in sociability elicited by S-SDFC was not due to gross changes in motor or exploratory activity during social testing, but rather due to their disrupted preference for the mouse relative to the toy.

All groups exhibited a similar latency to emerge from the dark box (Fig. 1i), however, there was a significant main effect of stress on the time spent in the dark compartment (Fig. 1j). Together, these findings revealed a specific deficit in sociability induced by S-SDFC. Given that no interaction effects were found in the anxiety-related paradigms, the disruption in social interaction was not likely due to stress-related anxiety occluding the motivation to socially interact.

In the stress-enhanced paradigms, female S-FC mice froze similarly at both retrieval tests, whereas freezing in S-SDFC mice was state-dependent, with significantly greater freezing during the GBX test when compared to the VEH test (Fig. S4a, b). However, unlike males, all female groups exhibited significant sociability (Fig. S4c). There were no significant differences between groups in the distance traveled (Fig. S4d) or in total exploration during sociability (Fig. S4e). S-SDFC significantly increased the latency to emerge from the dark box during anxiety-related testing (Fig. S4f), however, the time spent in the dark box was similar across groups (Fig. S4g). Together, these data show that the effects of stress-related memories on social interactions are sexually dimorphic and especially disruptive in males.

### Characterization of Oxtr-HI and their projections

**Fig. 2:**
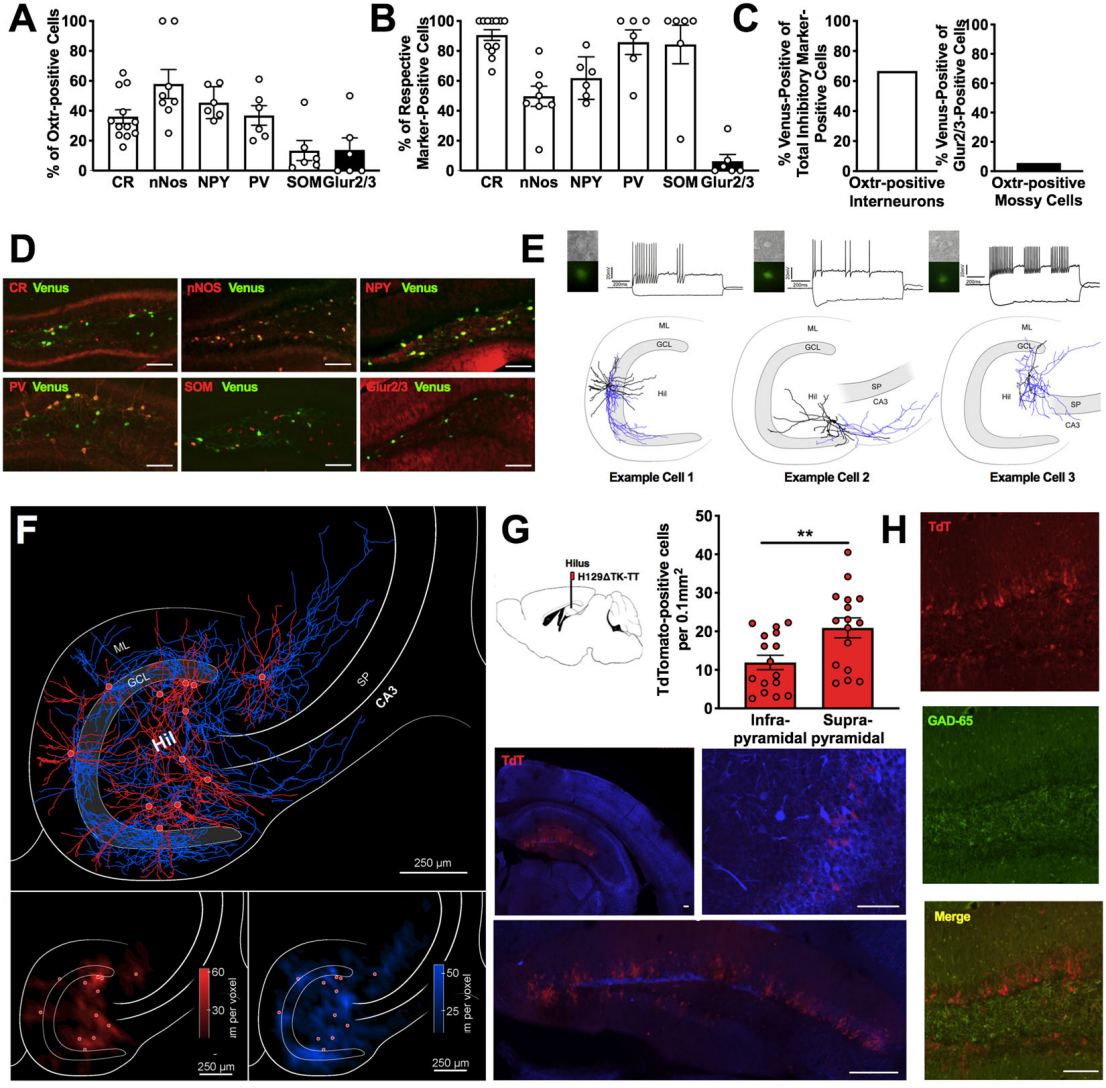
Oxtr-positive hilar neurons are a diverse population of interneurons which project to DGGC. **a** Quantification of Oxtr colocalization with respective marker, expressed as a percentage of total Oxtr-positive cells. *n* = 6CR/4nNOS/3NPY/3PV/3SOM/3Glur2/3 mice per group. **b** Quantification of Oxtr colocalization with respective marker, expressed as a percentage of marker-positive cells *n* = 3–6 mice per group. **c** Quantification of Oxtr colocalization with total interneuron markers or mossy cell marker, expressed as a percentage of total interneuron marker-positive or Glur2/3-positive, respectively. **d** Representative images of Oxtr and respective markers of interneurons and mossy cell coexpression in the DG. **e** Oxtr-positive hilar neurons were identified visually and recorded in voltage-clamp configuration mode. Heterogeneous firing patterns were observed. **f** Summary plot of all reconstructed axonal (blue) and dendritic (red) distribution patterns of Oxtr-HI merged into a map of the hippocampus. Note axonal arborization covering the entire DG. Also notice the presence of individual collaterals exiting the DG and projecting to area CA3 of the hippocampal formation. Bottom inset, quantitative analysis of the dendritic, bottom left, and axonal, bottom right, domains, as shown in contour plot indicating the density of either the dendritic and axonal segments with respect to their position in the hippocampus. Note that the dendritic domains are mainly restricted to the layer of the origin of somata (red dots). Oxtr-HI axons show a distributed density pattern with projections into the dentate granule cell layer, stratum moleculare, and CA3 stratum oriens, stratum lucidum, stratum radiatum, and stratum lacunosum moleculare. Voxel size 50 μm^3^. **g** Representative widefield florescent microscopy images showing TdT-positive cell bodies following injection of Cre-dependent transsynaptic anterograde tracer in the hilus of Oxtr-Cre mice. Note the presence of TdT-positive cell bodies in the DG GCL, and modest expression in the hilus and CA3 stratum pyramidale. Quantification of TdT-positive neurons in the suprapyramidal and infrapyramidal DG blades, respectively. A difference was observed, with significantly more TdT-positive cell bodies in the suprapyramidal blade. *n* = 8 mice; *t* = 2.836, df = 30, *P* = 0.0081. **h** TdT-positive cell bodies in the GCL were rarely colocalized with GAD65 positive neurons. Scale bars: 250 μm. **P < 0.01. ML moleculare layer, GCL granule cell layer, Hil Hilus, SP stratum pyramidale.

The neurochemical identity of Oxtr-HI was examined in a reporter mouse that expresses Venus driven by the Oxtr promoter^31^. Immunostaining against various markers for inhibitory interneurons and Glur2/3 for mossy cells^41^ revealed that the population of Oxtr-positive hilar neurons consists of calretinin (CR)-, neuronal nitric oxide synthase (nNOS)-, neuropeptide Y (NPY)-, parvalbumin (PV)-, and somatostatin (SOM)-expressing interneurons, as well as Glur2/3-expressing mossy cells in males (Figs. 2a, d and S5a, b, e) and females (Fig. S5c, d, f, g). Almost 100% of CR-, PV-, and SOM-expressing interneurons were found to be Oxtr-positive (Fig. 2b). Furthermore, we found that across all cellular subtypes addressed, Oxtr colocalized with the majority of interneurons (66.7%, Fig. 2c). In contrast, Oxtr colocalized only with a small proportion of mossy cells (5.6%). Together, these results demonstrated that Oxtr-positive hilar neurons are a heterogeneous population, primarily consisting of interneurons. To determine the local connectivity of Oxtr-HI, we mapped their projections using two complementary mapping approaches, taking advantage of the Oxtr-reporter mouse line, as well as an Oxtr-driven Cre mouse line. In the first mapping approach, hippocampal slices from Oxtr-reporter mice were obtained and Oxtr-HI were filled with biocytin and firing patterns were recorded (Fig. 2e). Both the recovered anatomy and the firing patterns supported our finding that Oxtr-HI are a diverse population. Quantitative analysis of the dendritic (Fig. 2f, bottom left) and axonal domains (Fig. 2f, bottom right), as shown in a contour plot indicating the density of axonal segments with respect to their position in the hippocampus, allowed us to classify sites of projection. Axonal collaterals originating from the hilar population were observed throughout the DG and CA3. In the DG, axons were found throughout all three laminae, including stratum moleculare, the granule cell layer (GCL), and the plexiform layer/hilus. Collaterals within CA3 were observed in stratum oriens, stratum lucidum, stratum radiatum, and stratum lacunosum moleculare. In the second approach, we examined the synaptic connectivity of dentate-projecting Oxtr-HI using a recently developed TdT transsynaptic anterograde tracer^42^. Oxtr-Cre mice were injected with a Cre-dependent H129ΔTK-TT viral vector, into the hilus, and the tissue was collected 24 h after infection. TdT signals were the strongest in the DG GCL (Fig. 2g) with sparse signals also detected in the hilus (Fig. 2g, bottom inset) and CA3 (Fig. 2g, right inset). TdT signals did not colocalize with GAD65, indicating that DG granule cells (DGGC), rather than GCL interneurons, receive afferents from Oxtr-HI (Fig. 2h). Analysis across DG blades demonstrated that there were significantly more TdT-positive neurons in the suprapyramidal blade, as compared to the infrapyramidal blade (Fig. 2g). Together, these findings demonstrate that the primary efferents of Oxtr-HI are DGGC in the suprapyramidal blade.

### Chemogenetic inactivation of Oxtr-HI ameliorates the sociability deficit elicited by S-SDFC

**Fig. 3:**
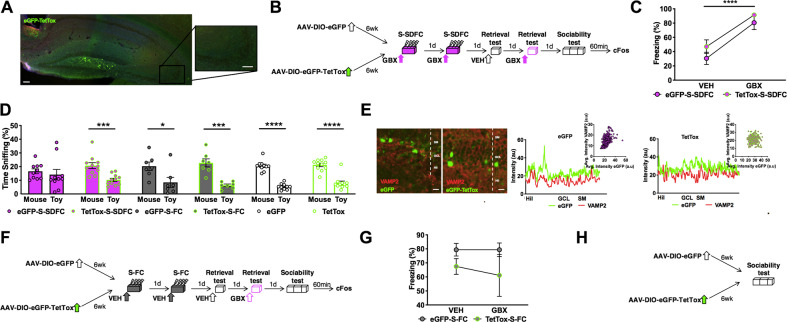
Inactivation of Oxtr-HI ameliorates the disruptive effects of stress-related memory on sociability. **a** Expression of Cre-dependent tetanus toxin for inactivating synaptic transmission in Oxtr-HI. Inset: CA3. Scale bars: 100 μm. **b** Schematic outline of the behavioral paradigm. Oxtr-Cre mice were infused with TetTox or control virus eGFP. Five and a half weeks later, mice were implanted with cannula targeting the DG. Three days later, mice were injected i.h. with GBX before S-SDFC (eGFP-S-SDFC and TetTox-S-SDFC groups, respectively). On alternating days, mice were tested for memory retrieval on VEH or GBX. Following memory retrieval tests, mice were subsequently tested for sociability. **c** During memory retrieval tests, eGFP-S-SDFC and TetTox-S-SDFC mice froze significantly less in the presence of VEH than in the presence of GBX. *n* = 10eGFP-SDFC/10TetTox-SDFC mice per group; retrieval drug *F*_(1,19)_ = 43.32, *P* < 0.0001; virus *F*_(1,19)_ = 2.416, NS; interaction *F*_(1,19)_ = 0.1499, NS. **d** During sociability, eGFP-S-SDFC exhibited no preference between the mouse and toy, whereas TetTox-S-SDFC mice displayed a significant preference for the mouse. *n* = 10eGFP-SDFC mice *t* = 0.5769, df = 18, *P* = 0.5712; *n* = 11TetTox-SDFC mice *t* = 4.275, df = 20, *P* = 0.0004. All control both groups exhibited a significant preference between mouse and toy. *n* = 7eGFP-S-FC mice *t* = 2.484, df = 12, *P* = 0.0287; *n* = 7TetTox-S-FC mice *t* = 5.303, df = 12, *P* = 0.0002; *n* = 10eGFP mice *t* = 10.67, df = 18, *P* < 0.0001; 11TetTox mice *t* = 6.948, df = 20, *P* < 0.0001. **e** Confocal images showing eGFP-TetTox or eGFP (green) and VAMP2 (red) immunostaining in the DG. The florescent intensity of eGFP and VAMP2 per pixel was significantly correlated in the eGFP group, but not in the TetTox group. *n* = 3eGFP mice; mouse 1 *r* = 0.4904, *P* < 0.0001 (plots shown); mouse 2 *r* = 0.2584, *P* < 0.0001; mouse 3 *r* = 0.4299, *P* < 0.0001; *n* = 3TetTox mice; mouse 1 *r* = −0.01018, *P* = 0.8541 (plots shown); mouse 2 *r* = 0.08578, *P* = 0.1269; mouse 3 *r* = −0.07788, *P* = 0.0692. **f** Schematic outline of the control experiment. Oxtr-Cre mice were infused with TetTox or eGFP and implanted with cannula, as before. Mice were injected i.h. with VEH before S-FC (eGFP-S-FC and TetTox-S-FC groups, respectively). On alternating days, mice were tested for memory retrieval on VEH or GBX and were subsequently tested for sociability. **g** During memory retrieval tests, both groups froze similarly in the presence of VEH or GBX. *n* = 7eGFP-S-FC/7TetTox-S-FC mice per group; retrieval drug *F*_(1,12)_ = 0.1389, NS; virus *F*_(1,12)_ = 2.971, NS; interaction *F*_(1,12)_ = 0.1389, NS. **h** Schematic outline of the behavioral paradigm. Oxtr-Cre mice were infused with eGFP or TetTox (eGFP and TetTox groups, respectively). Six weeks later, mice were tested for sociability. Scale bars: 10 μm. *P* < 0.0001. *P < 0.05; ***P < 0.001; ****P < 0.0001. Hil hilus, GCL granule cell layer, SM stratum moleculare.

We next sought to determine whether DGGC-projecting Oxtr-HI drive the sociability effects of S-SDFC. To conditionally inactivate this interneuron population, Oxtr-Cre mice were infused with AAV-DJ-CAG-DIO-TetTox-eGFP (TetTox) or control virus AAV-DJ-CMV-DIO-eGFP (eGFP). The virus expression was localized to the hilus (Fig. 3a) and did not spread to CA3 pyramidal neurons, which also express Oxtr^31^ (Fig. 3a, inset). After allowing five and a half weeks for viral expression, mice were implanted with cannula targeting the DG. Three days later, TetTox and eGFP mice were trained in the S-SDFC paradigm, followed by memory retrieval and sociability testing, as schematically outlined in Fig. 3b. During memory retrieval tests, eGFP-S-SDFC and TetTox-S-SDFC mice showed robust state-dependent freezing, when freezing was compared between the VEH and GBX tests (Fig. 3c). These results demonstrated that chemogenetic inactivation of Oxtr-HI did not disrupt state-dependent contextual fear. Consistent with our earlier experiment, eGFP-S-SDFC mice lacked preference for the mouse relative to the toy (Fig. 3d), whereas the TetTox-S-SDFC group displayed a significant social preference. We performed two additional control experiments to determine whether inactivation of Oxtr-HI by TetTox affects social interactions independently of S-SDFC. In the first experiment, effects of eGFP and TetTox were examined in the S-FC paradigm. Oxtr-Cre mice were infused with eGFP or TetTox and five and a half weeks later were implanted with cannula. Three days later, mice were trained with S-FC, followed by memory retrieval and sociability testing. Tissue was collected 1 h after sociability, as schematically outlined in Fig. 3f. The eGFP-S-FC and TetTox-S-FC groups showed similar freezing during VEH and GBX tests (Fig. 3g), indicating that inactivation of Oxtr-HI had no impact on memory. During sociability testing, both eGFP-S-FC and TetTox-S-FC groups displayed a significant preference for the mouse over the toy (Fig. 3d). In the second experiment, Oxtr-Cre mice were infused with TetTox or eGFP and 6 weeks later were tested for sociability without prior fear conditioning (Fig. 3h). During sociability, both groups demonstrated a significant preference for the mouse over the toy (Fig. 3d). These experiments demonstrated that inactivation of Oxtr-HI specifically ameliorated the sociability deficit following S-SDFC, without resulting in a generalized enhancement of sociability. Chemogenetic inactivation with TetTox, which is known to cause robust inhibition of neuronal output^43–45^, was confirmed by the disrupted correlation of VAMP2 and eGFP throughout the DG lamina of individual TetTox mice, when compared to individual eGFP mice (Fig. 3e).

To identify whether the observed effects are dependent upon the Oxtr, itself, Oxtr^loxP/loxP^ mice were infused with AAV2-CMV-eGFP-Cre (Cre). After allowing five and a half weeks for viral expression, mice were implanted with cannula targeting the DG. Three days later, mice were trained in the S-SDFC paradigm (Cre-S-SDFC), followed by memory retrieval and sociability testing, as schematically outlined in (Fig. S6a). Consistent with our earlier experiment, Cre-S-SDFC mice displayed robust state-dependent freezing (Fig. S6b), and did not display a preference for the mouse when compared to the toy (Fig. S6c). Viral-mediated Oxtr knockdown with Cre was confirmed by Cre expression and reduction of Oxtr mRNA in the DG of Cre mice, as compared to GFP mice see ref. ^46^, and data not shown. These experiments demonstrate that Oxtr-HI regulate amelioration of sociability deficits following S-SDFC through mechanisms independent of the Oxtr. Application of GBX (10 µM) induced from baseline a tonic current of −3.05 ± 0.35 pA/pF in DGGC, which was blocked (98 ± 0.13% reduction) by co-application of the GABA_A_R antagonist gabazine (12.5 mM). When the same experiment was performed in Oxtr-HI, significantly smaller GBX-induced currents were observed (−0.82 ± 0.17 pA/pF). However, no differences were observed in the responsivity of DGGC (−2.53 ± 0.20 pA/pF) or Oxtr-HI (−1.34 ± 0.47 pA/pF) to GBX between genotypes (Fig. S6d–f). These experiments demonstrate that in the DG, GBX mediates its tonic effects primarily upon DGGC through mechanisms independent of the Oxtr.

### Patterning of DG and CA3 activity in response to social interactions

**Fig. 4:**
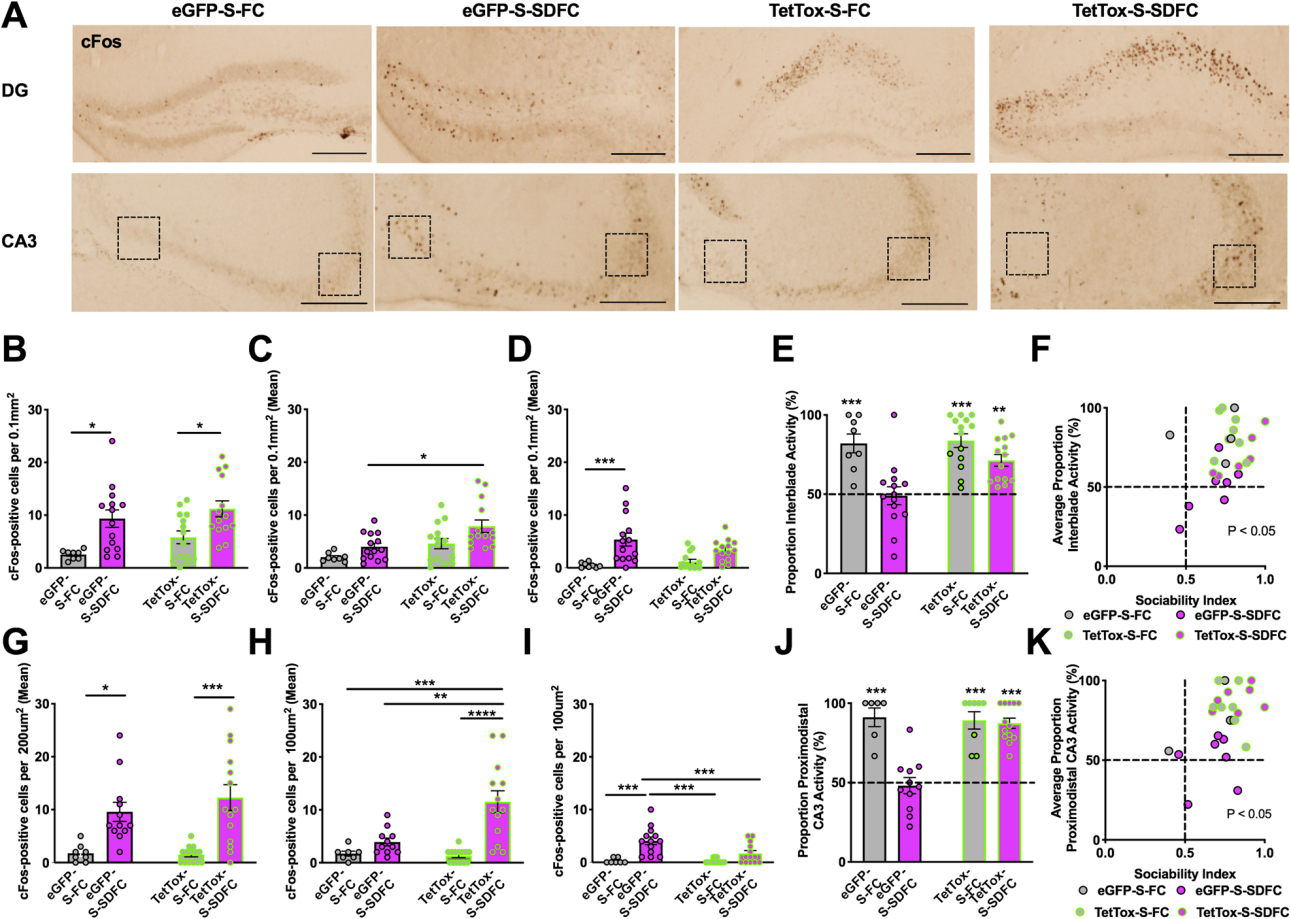
Segregated organization of sociability information in the DG and CA3. **a** Representative light microscopy images showing cFos immunostaining in the DG (top row) and CA3 (bottom row) in GFP-S-FC, GFP-S-SDFC, TetTox-S-FC, and TetTox-S-SDFC groups, respectively. Scale bars: 250 μm. **b** Quantification of cFos-positive neurons in the DG of Oxtr-Cre mice infused with virus GFP or TetTox and conditioned with S-FC or S-SDFC procedure. In total DG cFos, significant differences were observed between S-FC and S-SDFC groups, but not between GFP-S-SDFC mice and TetTox-S-SDFC mice. *n* = 4eGFP-S-FC/7eGFP-S-SDFC/7TetTox-S-FC/7TetTox-S-SDFC; conditioning drug *F*_(1,46)_ = 16.84, *P* = 0.0002; virus *F*_(1,46)_ = 2.990, NS; interaction *F*_(1,46)_ = 0.2155, NS. **c** In the suprapyramidal blade, TetTox-S-SDFC mice had significantly more cFos-positive neurons than GFP-S-SDFC mice. *n* = 4–7 mice per group; conditioning drug *F*_(1,46)_ = 7.292, *P* = 0.0097; virus *F*_(1,46)_ = 11.01, *P* = 0.0018; interaction *F*_(1,46)_ = 46.80, NS. **d** In the infrapyramidal blade, GFP-S-SDFC mice had significantly more cFos-positive neurons than GFP-S-FC and TetTox-S-FC mice. *n* = 4–7 mice per group; conditioning drug *F*_(1,46)_ = 18.62, *P* < 0.0001; virus *F*_(1,46)_ = 0.6615, NS; interaction *F*_(1,46)_ = 2.831, NS. **e** A proportion of interblade activity was calculated in which the area normalized number of cFos-positive neurons in the suprapyramidal blade was divided by the normalized number of cFos-positive neurons in both blades. This proportion revealed a significance difference between GBX-S-SDFC mice and all other groups. *n* = 4–7 mice per group; conditioning drug *F*_(1,46)_ = 6.626, *P* = 0.0130; virus *F*_(1,46)_ = 23.8, *P* < 0.001; interaction *F*_(1,46)_ = 4.831, *P* = 0.0330. **f** Correlation between sociability index and average proportion of interblade activity. *n* = 25, *r* = 0.4657, *P* = 0.0190. **g** In total CA3 cFos, significant differences were observed between GFP-S-SDFC mice and GFP-S-FC and TetTox-S-FC mice, but not between GFP-S-SDFC mice and TetTox-S-SDFC mice. *n* = 4–7 mice per group; conditioning drug *F*_(1,43)_ = 28.59, *P* < 0.0001; virus *F*_(1,43)_ = 0.4954, NS; interaction *F*_(1,43)_ = 0.7183, NS. **h** In distal CA3, significant changes were found in the TetTox-S-SDFC group, as compared to GFP-S-SDFC mice. *n* = 4–7 mice per group; conditioning drug *F*_(1,41)_ = 17.66, *P* = 0.0001; virus *F*_(1,41)_ = 5.89, *P* = 0.0197; interaction *F*_(1,41)_ = 7.385, *P* = 0.0096. **i** In proximal CA3, GFP-S-SDFC mice had significantly more cFos-positive neurons than all other groups. *n* = 4–7 mice per group; conditioning drug *F*_(1,42)_ = 28.56, *P* < 0.0001; virus *F*_(1,42)_ = 6.887, *P* = 0.0121; interaction *F*_(1,42)_ = 6.184, *P* = 0.0169. **j** A proportion of proximodistal CA3 activity was calculated in which the number of cFos-positive neurons in distal CA3 frame was divided by the number of cFos-positive neurons in both proximal and distal CA3 frames. This proportion revealed a significance difference between GFP-S-SDFC mice and all other groups. *n* = 4–6 mice per group; conditioning drug *F*_(1,34)_ = 19.09, *P* = 0.0001; virus *F*_(1,34)_ = 13.18, *P* = 0.0009; interaction *F*_(1,34)_ = 16.0, *P* = 0.0003. **k** Correlation between sociability index and average proportion of proximodistal CA3 activity. *n* = 24, *r* = 0.5377, *p* = 0.0067.

To establish whether stress-related memories change the patterns of hippocampal activity during social interactions and determine whether inactivation of Oxtr-HI contributes to the generation of these patterns, we compared the level of cFos in Oxtr-Cre mice injected with eGFP or TetTox and trained with VEH or GBX (eGFP-S-FC, eGFP-S-SDFC, TetTox-S-FC, and TetTox-S-SDFC groups). Tissue was collected 1 h after sociability tests from the experiments outlined in Fig. 3b, f. Quantification of cFos-positive neurons was performed for total DG and CA3 and separately for each DG blade and proximal and distal CA3. The total number of cFos-positive neurons in the DG was significantly increased in both GBX groups (Fig. 4a, b), suggesting that stress-related memories encoded under enhanced tonic inhibition render the DG hyperexcitable in response to subsequent social stimuli. However, this could not account for the social deficits, which were only found in eGFP-S-SDFC, but not in TetTox-S-SDFC mice. We therefore quantified the cFos-positive nuclei by individual DG blades (Fig. 4c, d) and found that, all groups, except for the eGFP-S-SDFC group, showed an asymmetric increase of cFos in the suprapyramidal blade (Fig. 4e). This pattern was blunted in the eGFP-S-SDFC group, which showed a significantly increased cFos response in the infrapyramidal blade, as compared to the control eGFP-S-FC group (Fig. 4d). Accordingly, the sociability index significantly correlated with the ratio of cFos activity between blades (Fig. 4f), but not with total cFos activity in the DG or in individual blades (Fig. S7a–c). Similar patterning of cFos up-regulation was found in CA3, showing a significant distal/proximal segregation in the eGFP-S-FC, TetTox-S-FC, and TetTox-S-SDFC groups but not in the eGFP-S-SDFC group (Figs. 4g–k and S7d–f). Notably, the disrupted patterns of cFos activity were normalized after inactivation of Oxtr-HI, which restored the activity ratios in DG and CA3. These results suggest that sociability is correlated with patterned DG/CA3 activity, which is disrupted by the encoding of state-dependent stress-related memories. These patterns can be restored by inhibition of Oxtr-HI that preferentially regulate the DG activity of the suprapyramidal blade and, consequently, distal CA3.

### Chemogenetic inactivation of dorsal hippocampal-caudal LS projections ameliorates S-SDFC-induced sociability deficits

**Fig. 5:**
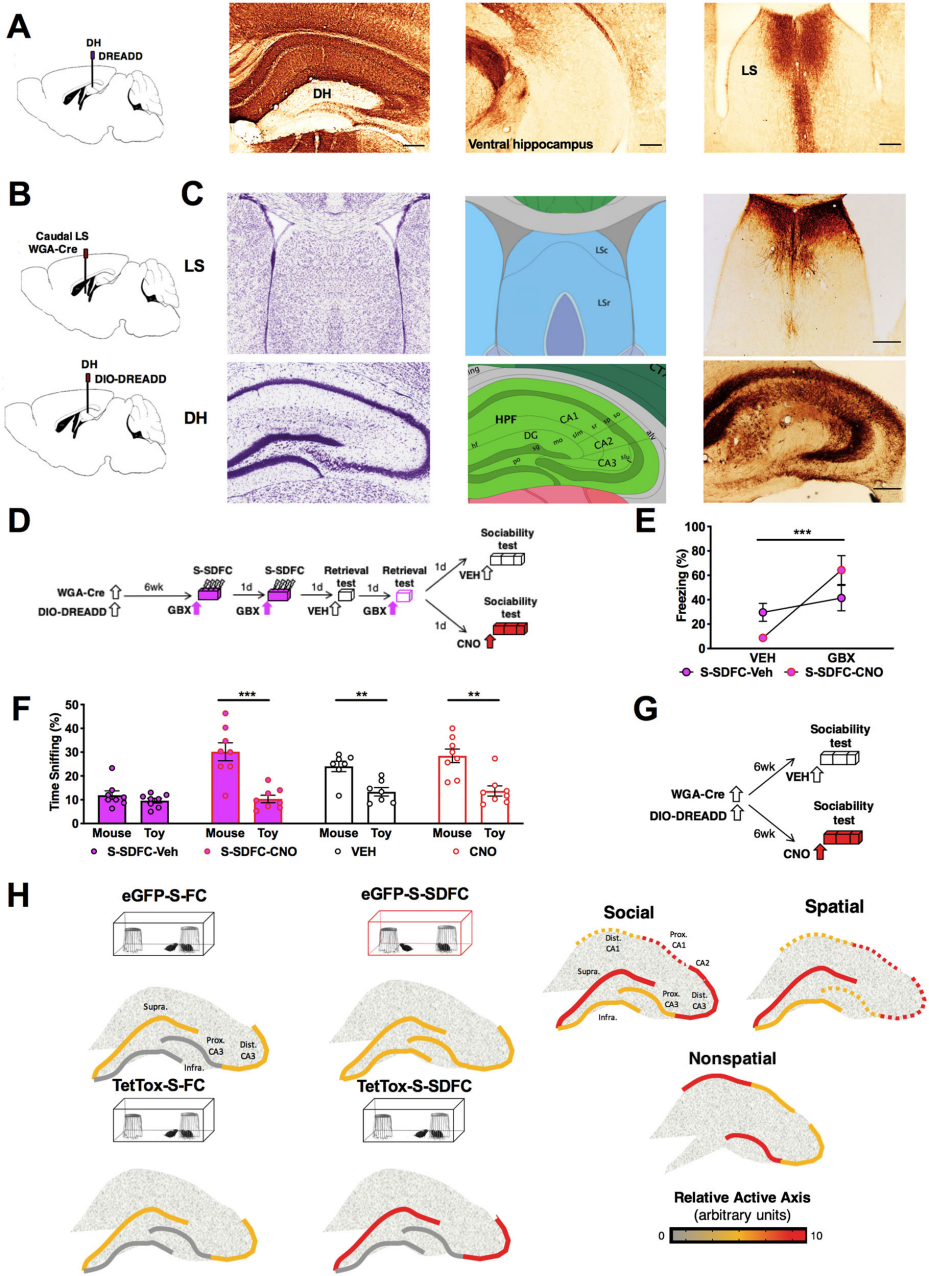
Inactivation of hippocampal-LS projections ameliorates disruptive social effects of S-SDFC. **a** AAV8-hSyn-hM4D(Gi)-mCherry encodes expression of mCherry (brown labeling) for mapping projections sites of hippocampal-septal projections. In the septum, dense projections in the caudal LS were observed. **b** Schematic illustration of the dual viral approach for inactivating synaptic transmission in LS-projecting hippocampal neurons. AAV8.EF1α.mCherry-WGA-Cre mediates expression of WGA-Cre. When this AAV infects a neuron, WGA-Cre is transneuronally transferred to connected neurons. Double-floxed inverted AAV8.hSyn-Hm4D(Gi)-mCherry encodes expression of mCherry for visualizing infected neurons and Hm4D(Gi) for conditionally blocking synaptic transmission. The coding region of the double-floxed inverted Hm4D(Gi) is not translated until Cre-recombinase flips the inverted coding region into the correct orientation. Scale bars: 250 μm. **c** Schematics of the neuroanatomical sites are on the left, images showing virus infection (mCherry signals, red) are on the right. LS-projecting hippocampal neurons were seen throughout the hippocampus, with particularly dense labeling in the CA3 field. Anatomic reference content was obtained from the Allen Brain Atlas^72^. **d** Schematic outline of the behavioral paradigm. Mice were infused with both viruses. Five and a half weeks later, mice were implanted with cannula targeting the DG. Three days later, mice were injected i.h. with GBX before a 2-day eight-shock contextual fear conditioning procedure. On alternating days, mice were tested for memory retrieval on VEH or GBX. Following memory retrieval tests, mice were injected i.h. with VEH or CNO before sociability testing (S-SDFC-VEH and S-SDFC-CNO groups, respectively). **e** During memory retrieval testing, S-SDFC-VEH and S-SDFC-CNO mice froze more in the presence of GBX compared to VEH. *n* = 8 mice per group; memory retrieval test drug *F*_(1,14)_ = 25.02, *P* = 0.0002; conditioning drug *F*_(1,14)_ = 0.008804, NS; interaction *F*_(1,14)_ = 10.69, *P* = 0.0056. **f** During sociability testing, S-SDFC-VEH mice did not display a significant preference between the mouse and toy, while S-SDFC-CNO mice did display a significant preference. *n* = 8 S-SDFC-VEH mice *t* = 1.113, df = 14, *P* = 0.2844; *n* = 8 S-SDFC-CNO mice *t* = 4.849, df = 14, *P* = 0.0003. Both control VEH and CNO mice displayed a significant preference between the mouse and toy. *n* = 7VEH mice *t* = 3.784, df = 12, *P* = 0.0026; *n* = 8CNO mice *t* = 4.128, df = 14, *P* = 0.0010. **g** Schematic outline of the behavioral paradigm. Mice were infused with both viruses. Five and a half weeks later, mice were implanted with cannula targeting the DG. Three days later, mice were i.h. with CNO before sociability testing. **h** Schematic of proposed theoretical model. (Left) In groups which exhibited sociability (three-compartment social behavior chamber, black outlines), a greater proportion of neurons was active in the suprapyramidal blade and distal CA3, as compared to the infrapyramidal blade and proximal CA3. In contrast, mice with disrupted sociability (three-compartment social behavior chamber, red outline) did not exhibit this asymmetric patterned activity, but instead displayed similar number of active cells between the DG blades and across proximodistal CA3. (Right) Models of hippocampal activity across the transverse axis in the regulation of social, spatial, and nonspatial tasks, respectively. Experimentally derived activity data (solid lines) are denoted from hypothetical activity (dashed lines) based upon known anatomical connectivity. Scale bars: 250 μm. **P < 0.01, ***P < 0.001. Alv. alveus, CA1. cornu ammonis 1, CA2. cornu ammonis 2, CA3. cornu ammonis 3, DG dentate gyrus, Dist. distal, fi. fimbria, hf. hippocampal sulcus, HPF. hippocampal formation, Infra. infrapyramidal granule cell blade, LSc. caudal lateral septum, LSr. rostral lateral septum, mo. startum moleculare, MS. medial septum, po. dentate gyrus polymorph layer, Prox. proximal, sg. granule cell layer, slm. stratum lacunosum moleculare, slu. stratum lucidem, so. stratum oriens, sp. stratum pyramidale, sr. stratum radiatum, Supra. suprapyramidal granule cell blade.

Given that SDFC is accompanied by increased activity in both the DG and LS^14^, we next investigated whether reducing activity in this pathway has the potential to ameliorate the S-SDFC-induced sociability deficits. To delineate LS projection sites stemming from the dorsal hippocampus (DH), wild-type mice were injected with AAV8-Hm4D(Gi)-mCherry viral vector into the DH. mCherry signals did not spread to the ventral hippocampus and were prevalent in the caudal LS (Fig. 5a). To conditionally inactivate the dorsal hippocampal-caudal LS projections, we used a dual virus approach, as illustrated in Fig. 5b, c. Wild-type mice were injected into the caudal LS with AAV8-EF1α-mCherry-WGA-Cre (WGA-Cre), and into the DH with Cre-dependent DREADD AAV8-hSyn-DIO-Hm4D(Gi)-mCherry (DIO-DREADD) five and a half weeks before implantation of cannula targeting the DG. Three days after cannula implantation, mice underwent S-SDFC, followed by off- and on-drug retrieval testing and sociability testing. Thirty minutes before sociability, mice were injected with either VEH or clozapine-N-oxide (CNO) via cannula targeting the DG, as schematically outlined in Fig. 5d. During memory retrieval tests, S-SDFC-VEH and S-SDFC-CNO mice showed impaired memory retrieval when tested on VEH, but intact retrieval when tested on GBX (Fig. 5e). During the sociability test, S-SDFC-VEH mice lacked a significant preference (Fig. 5f), however, such deficit was not found in mice when CNO was infused prior to sociability (S-SDFC-CNO group). These results demonstrate that inactivation of DH-caudal LS projections ameliorates the sociability deficit elicited by S-SDFC. To provide evidence that inactivation of these projections rescues S-SDFC-elicited sociability deficits, rather than enhancing sociability in general, we performed a control experiment in which S-SDFC was omitted. Wild-type mice were injected with dual WGA-Cre in the caudal LS and DIO-DREADD in the DH, as before. Five and a half weeks later, mice were implanted with cannula targeting the DG. Three days after cannula implantation, mice were infused with VEH or CNO prior to sociability testing (Fig. 5g). At sociability testing, both groups displayed a significant preference for the conspecific stimuli, as compared to the toy (Fig. 5f), which demonstrates that CNO did not enhance sociability nonspecifically. Together, these data provide evidence for a hippocampal efferent circuit to the caudal LS mediating social behavioral deficits.

We also examined whether activation of dorsal hippocampal-caudal LS projections is sufficient to disrupt sociability. To conditionally activate these projections, wild-type mice were injected into the DH with AAV8-hSyn-Hm3D(Gq)-mCherry 6 weeks before implantation of cannula targeting the caudal LS. Thirty minutes before sociability, mice were injected with either VEH or CNO via cannula, as schematically outlined in Fig. S8a, b. Tissue was collected 1 h after sociability testing. Virus expression was present throughout the DH (Fig S8c). During the sociability test, all groups displayed a significant preference for the conspecific stimuli, as compared to the toy (Fig. S8d). Activation of the caudal LS was confirmed by cFos analysis (Fig. S8e, f). Together, these results demonstrate that activation of DH-caudal LS projections, although necessary, does not seem to be sufficient to disrupt social behavior under the employed conditions.

## Discussion

Here we demonstrate that memories encoded under conditions of enhanced stress and elevated tonic inhibition result in robust social deficits in male mice. The encoding of such memories resulted in disruption of the patterned responses to social stimuli across the DG blades and CA3 subregions, and induced abnormal functioning of dorsohippocampal–caudal LS projections. The DG/CA3 activity and social interactions could be restored by inhibition of Oxtr-HI, an effect that was more likely due to their anatomical and functional connectivity to the suprapyramidal blade, rather than to Oxtr signaling.

We also report the greater susceptibility to stress-elicited social deficits in male mice compared to females, which may be due to observed sex difference in Oxtr-positive hilar mossy cells. These data support and extend previous findings, which indicate that important behavioral^47^ and neurobiological sex differences^48^ exist in most rodent stress models, including sex-specific differences in social behavior following stress^49,50^. Our data suggest that males are more sensitive to the physical footshock stressor in S-SDFC, which is consistent with findings that females respond inconsistently to physical stress^51^ and in some cases even respond with increased sociability^52^.

Interestingly, the disruptive effects of stress on social behavior were found when stress was delivered during increased hippocampal tonic inhibition. Although we used a pharmacological approach to ensure consistency of our manipulations, tonic inhibition can also be enhanced by stress. For example, the main stress mediators, such as corticosterone, can increase tonic inhibition either directly or indirectly through neurosteroid release, as an adaptive and protective mechanism from stress-induced anxiety and depression^53^. However, our findings suggest that the processing of intense stress under conditions of elevated tonic inhibition could have maladaptive consequences on social and anxiety-like behavior, as it has been previously found in models of chronic stress^54,55^. Our initial insight into the mediators of stress-induced social deficits identifies DG hyperactivity and disrupted patterning of activity as important contributing factors. It is not yet clear whether these changes are caused by enhancement of DGGC intrinsic activity or by persistent inhibition of DG hilar interneurons, which are known to disinhibit DGGC activity^56^. Both DGGC and DG interneurons, especially PV-positive and neurogliaform DG interneurons^57,58^, express high levels of the GABA_A_R δ-subunit, and could have been recruited in the memory circuit by GBX during S-SDFC.

Although the GCL has been canonically viewed as a functionally homogenous lamina whose total activity from both blades dictates the functional output, it is increasingly recognized that the DG blades are anatomically^59^ and functionally distinct^23,24,60–62^. Our findings contribute to this emerging view by demonstrating that social stimuli, similar to spatial and olfactory stimuli, are patterned within functionally discrete subdivisions in individual hippocampal subfields. On this basis, and based on our analysis of cFos activity in the DG and CA3, we propose that inactivation of Oxtr-HI ameliorates stress-induced sociability deficits through recovery of this asymmetrical suprapyramidal blade-distal CA3 pattern (model depicted in Fig. 5h, left). The asymmetrically patterned activity within a hippocampal subfield may retain its segregation as information is propagated downstream within the trisynaptic circuit, from anatomically connected proximal CA3 to distal CA1^23,26^. We therefore suggest that social information may subsequently be propagated toward CA2 and ventral proximal CA1 networks (Fig. 5h, right), whose efferents to the LS and other subcortical areas regulate social behavior^63–66^. Interestingly, the hippocampal activity patterns induced by social stimuli resembled those proposed for spatial but not nonspatial (olfactory) stimuli. This finding was surprising given the important role of olfactory stimuli in social interactions, however, it is possible that representations of social and olfactory stimuli are integrated into a “social context” within hippocampal efferent targets.

Our data support and extend previous findings regarding the diverse neurochemical identity of Oxtr-HI. In addition to the previously identified PV- and SOM-expressing Oxtr-HI^30,67^, we additionally report coexpression with markers for CR-, nNOS-, and NPY-positive interneurons, with few Oxtr-positive excitatory mossy cells. In light of our finding that Oxtr are expressed on the majority of hilar interneurons, thereby providing a pan-interneuronal marker, we were able to investigate the regulation of the DG by the broadly classified category of hilar interneurons. Interestingly, the preferential anatomical connectivity to the suprapyramidal blade appears to be the main determinant of the regulation of DG activity by Oxtr-HI. This is further supported by the lack of involvement of Oxtr in sociability^30,35,68^. In line with the behavioral findings, tonic inhibition in DGGC and Oxtr-HI was not affected by Oxtr knockout, suggesting that the local connectivity, rather than Oxtr expression, was the predominant determinant of the effects of Oxtr-HI on DG activity in response to social stimuli.

Alterations of local hippocampal activity are also expected to impact its efferent targets. Consistent with our earlier work identifying the importance of the caudal LS in the regulation of sociability^35^, we found that hippocampal efferents to this area were responsible, at least in part, for the social behavioral deficits observed in S-SDFC mice. Earlier findings suggest that this is most likely mediated through a CA3-fimbria-LS circuit based upon the known anatomical connectivity^69^ and documented role of CA3 and the fimbria in sociability^70,71^. However, possible roles of CA1 and especially CA2, which also projects to the LS^66^, cannot be ruled out.

Taken together, we identify cellular and circuit mechanisms in the hippocampus by which encoding of stress-related memories with restricted retrievability adversely affect social interactions. Inhibition of Oxtr-HI and the dorsohippocampal–LS pathway was identified as effective manipulations for normalization of patterned hippocampal activity and restoration of social interactions after stress.

## Supplementary information

Supplemental Material
