# Supplementary material for: Stress-related memories disrupt sociability and associated patterning of hippocampal activity: a role of hilar oxytocin receptor-positive interneurons
Source: Transl Psychiatry. 2020 Dec 12;10:428. doi: 10.1038/s41398-020-01091-y (PMC7733596; doi:10.1038/s41398-020-01091-y)
Supplement: Supplementary file 1 — Supplemental Material [file 41398_2020_1091_MOESM1_ESM.docx]

**Supplementary Information**

**Supplementary Materials and Methods**

**Mouse genotyping and housing**

All transgenic lines were maintained by heterozygous (Oxtr-reporter) or homozygous (Oxtr-Cre and Oxtr^loxP/loxP^) breeding in Northwestern University’s animal facility. Mice were bred, genotyped (using primers 5’-AGA GTG TCT CGT GTG GCA GAA G-3’, 5’ -GGA GTA GAA GGT GGC GCG AA-3’, 5’ -CCA CTG GCT TCT AAT GCA TG-3’, as reported by^(32)^. Typically, we obtained 4-6 litters/breeding cycle with 5-8 mice/litter with similar distribution of males and females. Mice used for experiments at the age of 8 weeks.

The mice were maintained under standard housing conditions (12/12 hr light dark cycle with lights on at 7am, temperature 20-22° C, humidity 30-50%) in our satellite behavioral facility. All behavioral experiments were performed between 7am and 7pm.

**Light-dark emergence**

Mice were placed in a three-sided dark covered box which was placed in the center of an open field illuminated by bright light (to increase open-field averseness). Animals’ position and motion were tracked by a top-mounted video camera connected to a computer and a DVD recorder. For analysis, videos were manually scored for latency to exit the box, as well as duration of time spent in the box by an investigator blind to animal treatment identity. Randomly selected videos were scored by two different blinded investigators.

**Stereotaxic surgeries and infusions of viral vectors and drugs**

Mice were anesthetized with isoflurane (induction 3-5%, maintenance 1-3%) for viral vector intracranial infusion or 1.2% tribromoethanol (vol/vol, Avertin) for cannula implantation. The viral vector carrying a construct coding for Cre-dependent Tetanus Toxin (AAV-DJ-CMV-DIO- eGFP-2A-TeNT, Stanford GVVC-AAV-71 3.6x10^12^ GC/mL), Cre (pAAV2-CMV-HI-eGFP- Cre-WPRE-SV40, Addgene, 105545-AAV2, 7 x 10^12^ GC/mL), or control eGFP (AAV-DJ-CMV- DIO-eGFP, Stanford GVVC-AAV-12, 5.9x10^12^ GC/mL) or GFP (pAAV2-CAG-GFP, Addgene, 37825-AAV2, 8.2 x 10^12^ GC/mL), were bilaterally infused into the hilus (1.8 mm posterior, ± 1.0 mm lateral, 2.3 mm ventral to bregma). The viral vector carrying a construct for Cre-dependent transsynaptic anterograde tracer (NIH Center for Neuroanatomy with Neurotropic viruses, H129ΔTK-TT, 5.52x10^8^ pfu/mL) was also infused into the hilus, though unilaterally. Cre-independent DREADDs (AAV8-hSyn-HA-hM4D(Gi)-mCherry, Addgene, 4.26 x10^12^ GC/mL) were infused in the DH (1.7 mm posterior, ± 1.0 mm lateral, 2.1 mm ventral to bregma). Cre-dependent DREADD (AAV8-hSyn-DIO-hM4D(Gi)-mCherry, Addgene 44362, 2.5x10^13^ GC/mL) was also infused bilaterally into the DH. WGA-Cre (AAV8-EF1α-mCherry-WGA-Cre, UNC AV5869, 2.7x10^12^ GC/mL) was infused unilaterally into the caudal LS (0.38mm anterior, 0.0 mm lateral, and 2.85 mm ventral to bregma). Infusions were performed using an automatic microsyringe pump controller (Micro4-WPI) connected to a Hamilton microsyringe. The viral vectors were infused in a volume of 0.5 uL per hippocampal site or 0.4 uL per LS site, over 2 min, and syringes were left in place for 5 min prior to removal to allow for virus diffusion.

Bilateral 26 gauge guide cannulas (Plastics One) were placed in DG (1.8 mm posterior, ± 1.0 mm lateral, 2.5 mm ventral to bregma). Mice were allowed 6 weeks for virus expression prior to behavioral testing, or 24h for monosynaptic transsynaptic tracing. Drugs were injected i.h. at a volume of 0.25 µl per side at a rate of 0.5 µl min. Extrasynaptic GABA_A_R were activated by gaboxadol hydrochloride (0.5 µg per dentate, dissolved in artificial cerebrospinal fluid; Sigma Aldrich 85118-33-8) 20 min prior to SDFC or retrieval test. DREADDs were activated by CNO (0.35 µg/mL, dissolved in artificial cerebrospinal fluid; Sigma Aldrich, C0832) infused through the cannulas 30 min prior sociability testing.

**Immunohistochemistry**

For immunohistochemistry and immunofluorescence studies, mice were anesthetized with an i.p. injection of 240 mg/kg Avertin and transcardially perfused with ice-cold 4% paraformaldehyde in phosphate buffer (pH 7.4, 150 mL per mouse). Brains were removed and post-fixed for 24h in the same fixative and then immersed for 24h each in 20% and 30% sucrose in phosphate buffer. Brains were frozen and 50 μm sections were cut for use in free-floating immunohistochemistry with primary antibodies against against Parvalbumin (PV; 1:5,000, Swant, PVG-213), Neuropeptide Y (NPY; 1:1,000, Immunostar, 22940-1628001), Somatostatin (SOM; 1:1,000, Millipore Sigma, MAB354), Calretinin (CR; 1:4,000, Swant, CG1), Neuronal Nitric Oxide Synthase (nNos; 1:500, Millipore Sigma, ab5380), Glur2/3 (3 μg/mL, Chemichon, AB1506), GFP (1: 1:4000, Abcam, ab13970; or 1:1000, Abcam, ab1218), mCherry (1:1000, Abcam AB167453), GAD65 (1:400, Abcam ABN101), VAMP2 (1:500, Synaptic Systems Cat #104211), cFos (1:2000, ProteinTech, 26192-1-AP). All secondary antibodies were obtained from Vector (1:100, Biotinylated- goat anti-horse (BA-9500), rabbit anti-goat (BA-1000), rat anti-rabbit (BA-4001), chicken anti-goat (BA-9010), or mouse anti-goat (BA-9200) IgG. For light microscopy, signals were visualized with diaminobenzidine (Sigma, D4168) and mounted using Eukitt Mounting Medium (Fisher Scientific, 50-980-467). For immunofluorescence, signals were visualized with Rhodamine or Fluorescein (1: 62.5, Perkin Elmer, SAT702001EA or SAT701001EA) and mounted using Vectashield (Vector, H-1000) and observed with on a Leica microscope, an Olympus Fluoview FV10i confocal microscope, or a Nikon Ti2 widefield microscope. For immunohistochemistry following recording and intracellular filling with biocytin, brain slices were fixed in 0.1 M phosphate-buffered (PB, pH 7.4) solution containing 4% paraformaldehyde (for light microscopy only) at 4°C for 24h. They were then processed for light microscopy, as described elsewhere^(73)^. In brief, after incubation in ABC-Elite solution (Sigma Aldrich) overnight, slices were pre-incubated in 3’3-diaminobenzidine (Sigma Aldrich) and visualized by adding 0.025% H_2_O_2_ to the solution. The reaction was stopped when dendritic and axonal processes were clearly visible. After several washing steps in 0.1 M PB sections were, after brief post-fixation in osmium tetroxide (1–2 min), and embedded in Moviol (Hoechst AG).

**Preparation of acute brain slices**

Oxtr-reporter mice (P15–P21; n = 3) were deeply anesthetized as described above and then decapitated. Acute slices in the horizontal plane (350–400 μm in thickness) were prepared using a Leica VT 1200S vibratome (Leica Microssystems, Wetzlar, Germany). Slices were cut in ice–cold ‘cutting’ artificial cerebrospinal fluid (ACSF) of the following composition (in mM): 130 NaCl, 24 NaHCO_3_, 3.5 KCl, 1.25 NaH_2_PO_4_, 1 CaCl_2_, 2 MgCl_2_, 10 glucose saturated with 95 % O_2_–5 % CO_2_ at pH 7.4. They were transferred in a storage chamber containing ‘recording’ ACSF (see above, with CaCl_2_ increased to 2 mM and MgCl_2_ reduced to 1 mM) and were allowed to adjust to room temperature (20–23 °C) for 30 min prior to recording.

**Visual identification of Oxtr-positive cells**

Slices were placed in the recording chamber under an upright Olympus microscope. Fluorescence of venus-containing Oxtr-expressing cells was excited by a Prizmatrix UHP 460nm LED (Prizmatix Ltd.) and visualized using a Andor Zyla 4.2 camera (Andor Technology Ltd.). Oxtr-expressing cells were visually identified at 600x magnification first by fluorescence imaging and subsequently by oblique contrast microscopy as well as their location in the hilus of the DG.

**Electrophysiological recordings and data analysis**

For morphological reconstruction experiments, pipettes were pulled from borosilicate thin glass capillaries, filled with filtered intracellular solution, with a final resistance of ~3 MΩ. The intracellular pipette solution contained (in mM) 125 K-methylsulfate, 10 NaCl, 0.3 GTP-Na, 4 ATP-Mg2, 16 KHCO_3_ and 0.3–0.5% biocytin, equilibrated with 95% O_2_, 5% CO_2_ to pH 7.3. For morphological analysis 1–2 mg/ml biocytin (Sigma Aldrich) was added routinely to the internal solution. During recording, slices were continuously superfused with ‘recording’ ACSF (see above). Membrane properties and firing characteristics of interneurons were determined in voltage- and current-clamp configuration. For tonic inhibition experiments, the intracellular pipette solution contained (in mM) 125 CsCl, 10 NaCl, 16 KHCO_3_, 4 ATP-Mg, 0.3 GTP-Na, and 10 QX314-Cl equilibrated to pH 7.3. Recordings were carried out using a Multiclamp 700B amplifier (Molecular Devices). Series resistances were balanced via a bridge circuit in the current-clamp mode. Data were filtered at 3 kHz and digitized at 10–20 kHz using a Digidata 1550A board and the Clampex 9 program suite (Molecular Devices) and stored on a hard disk of a desktop computer. All recordings and measurements were carried out at 29–31°C. For tonic current experiments, the baseline current was analyzed, as previously described^(74)^. Briefly, by generating an all-points histogram and fitting a Gaussian distribution to the positive side of the histogram which is uncontaminated by negative-going inhibitory post synaptic currents that will skew the distribution. Then, the means of the fitted Gaussians were used to define the holding currents before and after drug application (GBX, 10 μM, Sigma Aldrich, C85118-33-8; Gabazine SR 95531 hydrobromide, 12.5 μM Tocris, 1262). Tonic currents were also normalized to the cell capacitance (pA/pF) to allow comparison between DGGC and Oxtr-HI.

**Morphological reconstructions of biocytin-filled Oxtr-positive cells**

Only neurons for which a complete physiological analysis was made and that had no obvious truncation of their dendritic and axonal profiles were used for qualitative analysis of their morphology. Representative examples were reconstructed using the Neurolucida software (MicroBrightfield) on a Zeiss Axioskop 2 (Carl Zeiss GmbH).

**Axonal and dendritic density maps**

2-dimensional (2D) maps of dendritic and axonal “segment-length density” were constructed using the computerized 2D reconstructions (for details see 75). For proper alignment of the cell-specific density all reconstructed Oxtr-expressing cells were projected in a 2D plane and centered to their axonal initial segment. For the “region-specific density” plots cells were aligned into a scheme of a hippocampal slice (horizontal plane) with respect to the relative soma and arborization position. Then axonal and dendritic segment-length was measured in a 50 µm by 50 μm Cartesian grid, yielding into a raw density map. Continuous 2D density functions were constructed using bicubic interpolation in Mathematica 7 (Wolfram Research).

**RNA extraction and quantitative PCR (qPCR)**

Mice were killed by cervical dislocation. Brains were dissected and the DG was collected using a brain matrix. Tissue was homogenized in lysis buffer with β-mercaptoethanol and frozen in liquid nitrogen. RNA was extracted using miRCURY total RNA isolation kit (Exiqon), reversely transcribed and subjected to real-time PCR using SYBR Green master mix (Applied Biosystems) and primers for Oxtr or mouse hypoxanthine phosphoribosyl transferase 1 (mHprt1) as an internal control. For Oxtr, the forward primer was 5’-GGA GCG TCT GGG ACG TCA AT-3” and the reverse primer was 5’-AGG AAG CGC TGC ACG AGT T-3’. For mHprt1, the forward primer was 5’-GGG CTT ACC TCA CTG CTT TC -3’ and reverse 5’-TCT CCA CCA ATA ACT TTT ATG TCC-3”. The level of Oxtr expression in Oxtr^loxP/loxP^ mice injected with Cre or GFP was normalized to mHprt1 and shown relative to the GFP control.

**Supplementary Figures**

**Fig. S1. Intensified-stress fear conditioning does not affect sociability behavior.** **a** Schematic outline of the behavioral paradigm. Mice were implanted with cannula targeting the dentate gyrus. Three days later, mice were injected i.h. with VEH before a four-day thirty-two-shock contextual fear conditioning procedure (S-FC32). Following conditioning, mice were tested for sociability. **b** During sociability testing, mice spent significantly more time sniffing the mouse compared to toy. n = 8 mice; t = 7.083, df = 14, P < 0.0001. **c** As compared to FC and S-FC mice, S-FC32 mice travelled similar distance during sociability. n = 5FC/10S-FC/8S-FC32 mice per group; stress F_(2,20)_ = 0.2634, NS. **d** As compared to FC mice, IS-FC mice spent significantly more time during sociability testing sniffing both stimuli. n = 9FC/10-S-FC/8S-FC32 mice per group; stress F_(2,24)_ = 12.9, P = 0.0002. (**) P < 0.01, (***) P < 0.001, (****) P < 0.0001.

**
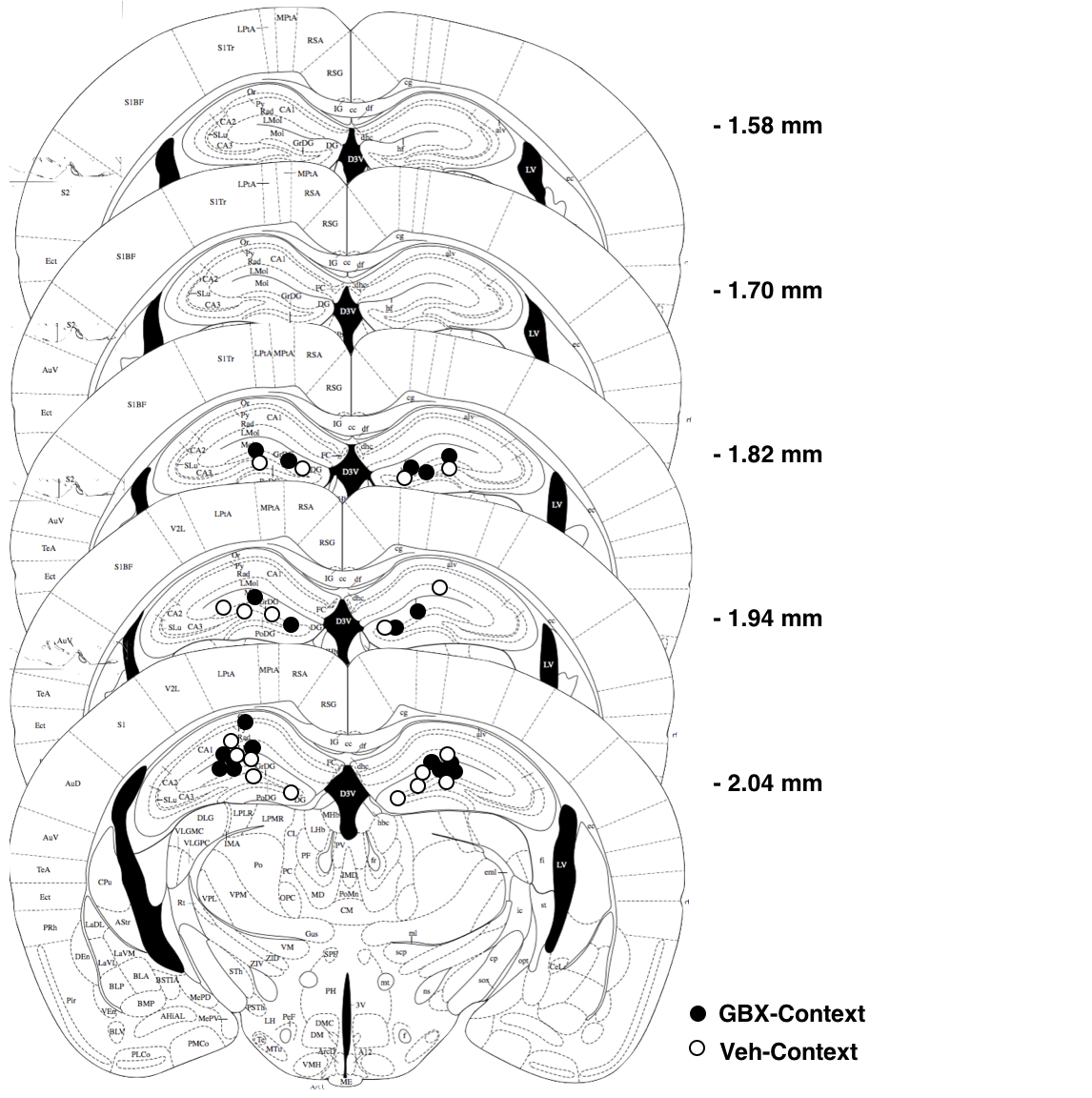
**

**Fig. S2. Representative DG cannula placements.** Cannula placement in one experiment to show consistency of DG localization. Anatomic reference content was obtained from the Allen Brain Atlas^(72)^.

**
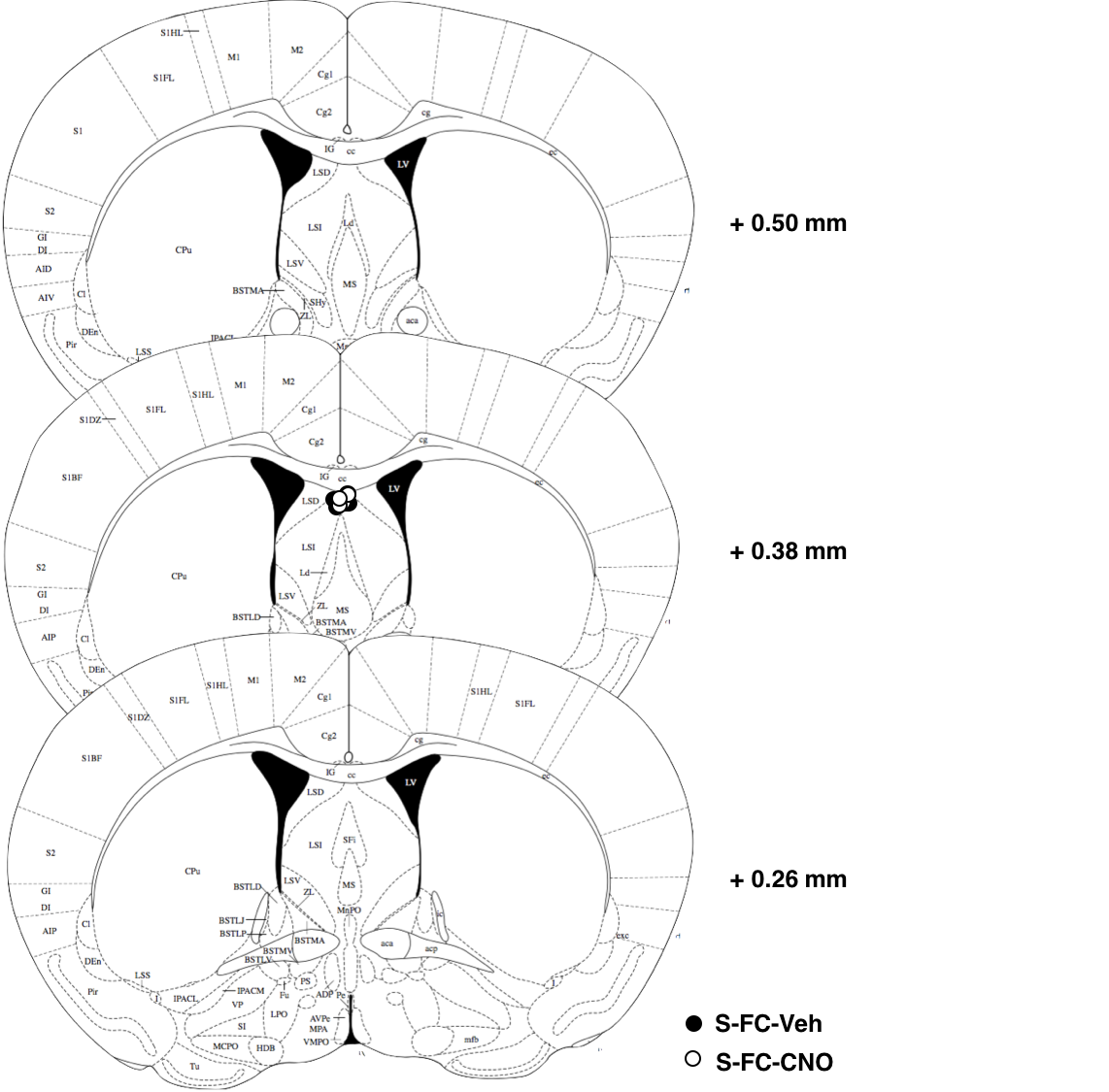
**

**Fig. S3. Representative caudal LS cannula placements.** Cannula placement in one experiment to show consistency of caudal LS localization. Anatomic reference content was obtained from the Allen Brain Atlas^(72)^.

**Fig. S4. Stress-related memory does not affect sociability in females.** **a** During the memory retrieval tests, FC mice froze significantly less at the GBX test than at the VEH test. In contrast, SDFC mice froze significantly more in the presence of GBX than in the presence of VEH. n = 11FC/9SDFC mice per group; conditioning drug F_(1,18)_ = 2.611, NS; memory retrieval test drug F_(1,18)_ = 4.753, P = 0.0428; interaction F_(1,18)_ = 36.40, P < 0.0001. **b** During the memory retrieval tests, S-FC mice froze similarly across tests. S-SDFC mice froze significantly less in the presence of VEH than in the presence of GBX. n = 7S-FC/10S-SDFC mice per group; conditioning drug F_(1,15)_ = 0.9129, NS; memory retrieval test drug F_(1,15)_ = 1.716, NS; interaction F_(1,15)_ = 23.07, P = 0.0002. **c** During sociability, all female groups spent significantly more time with the mouse when compared with the toy. n = 18Veh-Context mice t = 3.405, df = 34, P = 0.0017; n = 11FC mice t = 3.459, df = 20, P = 0.0025; n = 8S-FC mice t = 2.371, df = 12, P = 0.0354; n 11GBX-Context mice t = 4.705, df = 20, P = 0.0001; n = 19SDFC mice t = 4.184, df = 36, P = 0.0002; n = 10S-SDFC mice t = 3.989, df = 18, P = 0.0009. **d** Neither the conditioning procedure, nor drug affected activity during sociability, as measured by distance traveled n = 14Veh-Context/11FC/7S-FC/11GBX-Context/17SDFC/10S-SDFC mice per group; conditioning drug F_(1, 63)_ = 6.509, P = 0.0132; stress F_(2, 63)_ = 2.249, NS; interaction F_(2, 63)_ = 0.2345, NS. **e** All groups exhibited similar total time spent sniffing both stimuli during sociability. n = 18Veh-Context/11FC/7S-FC/11GBX-Context/19SDFC/10S-SDFC mice per group; conditioning drug F_(1, 68)_ = 1.779, NS; stress F_(2, 68)_ = 2.238, NS; interaction F_(2, 68)_ = 0.9551, NS. **f** Latency to emerge from dark box during light-dark emergence testing was increased in SDFC and S-SDFC groups, as compared to the GBX-Context group. n = 11VEH-Context/11FC/7S-FC/11GBX-Context/11SDFC/8S-SDFC; conditioning drug F_(1, 53)_ = 1.805, NS; stress F_(2, 53)_ = 6.265, P = 0.0036; interaction F_(2, 53)_ = 1.268, NS. **g** All groups exhibited similar time in dark during light-dark emergence paradigm. n = 11VEH-Context/11FC/7S-FC/11GBX-Context/11SDFC/8S-SDFC mice per group; conditioning drug F_(1, 54)_ = 1.496, NS; stress F_(2, 54)_ = 1.026, NS; interaction F_(2, 54)_ = 1.837, NS. (*) P < 0.05, (**) P < 0.01, (***) P < 0.001, (****) P < 0.0001.


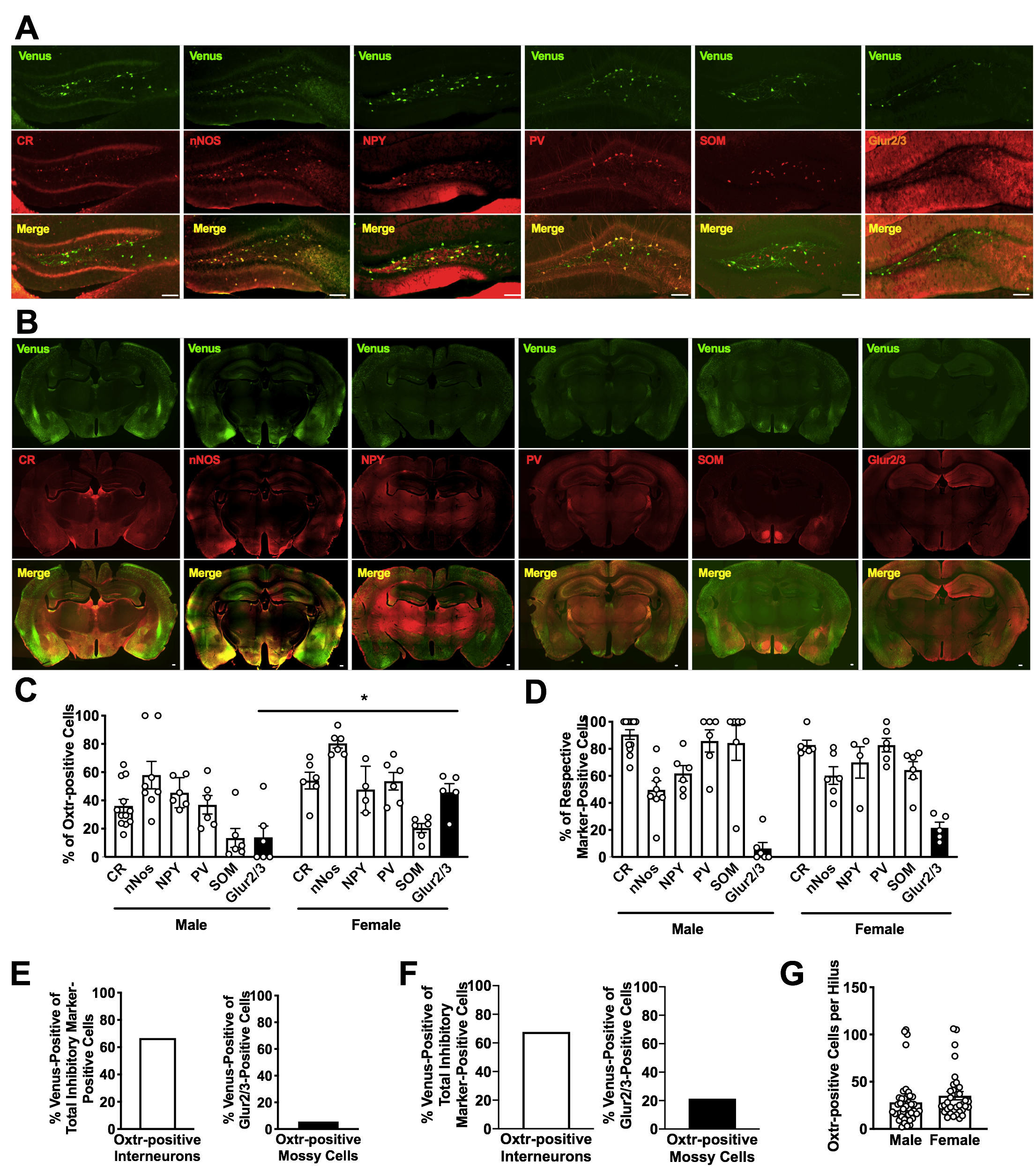
**Fig. S5.** **Characterization of sex-differences in Oxtr-positive neuronal populations in the hilus**. **a** Representative images of Oxtr and respective markers of interneurons and mossy cell expression in the dentate gyrus. **b** Representative images of Oxtr and respective markers of interneurons and mossy cell expression across the entire brain. (**c**) Quantification of Oxtr colocalization with respective marker, expressed as a percentage of total Oxtr-positive cells. n = 6CR/4nNOS/3NPY/3PV/ 3SOM/3Glur2/3 male mice per group and n = 3CR/3nNOS/2NPY/3PV/3SOM/3Glur2/3female mice per group; sex F_(1, 65)_ = 17.86, P < 0.0001; marker F_(5, 65)_ = 14.48, P < 0.0001; interaction . F_(5, 65)_ = 1.127, P = 0.3549. As there was an effect of sex, posthoc was run for sex differences. (**d**) Quantification of Oxtr colocalization with respective marker, expressed as a percentage of marker-positive cells n = 3-6 mice per group; marker F_(5, 65)_ = 30.01, P < 0.0001, sex F_(1, 65)_ = 0.007378, NS; interaction marker F_(5, 65)_ = 1.816, NS. (**e-f**) Quantification of Oxtr colocalization with total interneuron markers or Glur2/3 mossy cell marker, respectively, expressed as a percentage of total count in males (**e**) and females (**f**). (**g**) The number of Oxtr-positive neurons per hilus did not differ between sexes; t = 1.221, df = 75, NS. Scale bars, 250 μm. (*) P < 0.05.

**Fig. S6. Conditional knockdown of hilar Oxtr does not affect sociability behavior and knockout does not affect tonic inhibition. a** Schematic outline of the behavioral paradigm. Oxtr^loxP/loxP^ mice were infused with Cre. Five and a half weeks later, mice were implanted with cannula targeting the dentate gyrus. Three days later, mice were injected i.h. with GBX before S-SDFC (Cre-S-SDFC). On alternating days, mice were tested for memory retrieval on VEH or GBX. Following memory retrieval tests, mice were subsequently tested for sociability. **b** During memory retrieval tests, Cre-S-SDFC mice froze significantly less in the presence of VEH than in the presence of GBX. n = 7Cre-S-SDFC mice t = 3.742, df = 6, P = 0.0096. **c** During sociability testing, Cre-S-SDFC mice did not display a preference between mouse and toy. n = 7 mice t = 2.027, df = 12, P = 0.0655. **d** Quantifications of current levels during baseline (gray) and tonic currents in the presence of GBX (magenta), and GBX plus GBZ (yellow) using Gaussian fits as described in the methods section. Example from a DGGC-Oxtr^+/venus^ mouse. Note the development of a tonic current when 10µM GBX was applied, which was blocked by 12.5µM GBZ. **e** Same experimental design as in d, but examples from a different DGGC in an Oxtr^+/venus^ mouse, a DGGC in an Oxtr^venus/venus^ mouse, and Oxtr-HI in Oxtr^+/venus^ and Oxtr^venus/venus^ mice, respectively. **f** Quantification of the tonic currents (normalized by cell capacitance) were obtained by subtracting current levels under the different experimental conditions (baseline, GBX, and GBX plus GBZ). Significant differences in GBX-induced currents were observed in DGGC, as compared to Oxtr-HI. n = 24DGGC-Oxtr^+/venus^/25DGGC-Oxtr^venus/venus^/11Oxtr-HI-Oxtr^+/venus^/5Oxtr-HI-Oxtr^venus/venus^ cells per group; main effect of cell type F_(1,61)_ = 19.76, P < 0.0001 (GBX-Baseline); and main effect of cell type F_(1,61)_ = 17.98, P < 0.0001 (GBX-GBZ). (*) P < 0.05, (**) P < 0.01, (****) P < 0.0001.

**Fig. S7**. **The sociability index is not correlated with cFos in individual DGGC blades or proximal and distal CA3. a-e**, Correlation of sociability index with cFos-positive neurons in DG blades and CA3. n = 25. **a** Total cFos in both DG blades. r= 0.08934 P = 0.6711. **b** Suprapyramidal blade. r= 0.324 P = 0.1141. **c** Infrapyramidal blade. r= -0.2764 P = 0.1810. **d** Total cFos in both frames of distal and proximal CA3. r= 0.05228 P = 0.8040. (**e**) Distal CA3. r = 0.2311 P = 0.2663. **f** Proximal CA3. r = - 0.2734 P = 0.1861.

**Fig. S8. Activation of dorsal hippocampal-caudal LS projections is not sufficient to disrupt sociability in the absence of stress or S-SDFC. a-b,** Schematic outlines of the behavioral paradigm. Wildtype mice were infused AAV8-hSyn-Hm3D(Gq)-mCherry in the DH. Five and a half weeks later, mice were implanted with cannula targeting the caudal LS. Three days later, **a**, mice underwent sociability testing, or **b**, S-FC followed by sociability testing. Thirty minutes before sociability, mice were injected with either VEH or CNO via cannula targeting the caudal LS (VEH, CNO, S-FC-VEH, and S-FC-CNO groups, respectively). Tissue was collected 1h after sociability testing. **c** DH expression of AAV8-hSyn-Hm3D(Gq)-mCherry for activating synaptic transmission in caudal LS. Scale bar, 250 μm. **d** During the sociability test, all groups displayed a significant preference for the mouse. n = 9VEH mice t = 5.925, df = 16, P < 0.0001; n = 10CNO mice t = 8.231, df = 18, P < 0.0001; n = 11S-FC-VEH mice t = 4.033, df = 18, P = 0.0008; n = 9S-FC-CNO mice t = 9.427, df = 18, P < 0.0001. **e** Representative light microscopy images showing cFos immunostaining in the caudal LS in VEH and CNO groups, respectively. Scale bars 250 μm. **f** Quantification of cFos-positive neurons in the caudal LS of wildtype mice infused with virus AAV8-hSyn-Hm3D(Gq)-mCherry and tested for sociability with VEH or CNO. The CNO group had significantly more active neurons in the caudal LS. n = 7VEH/9CNO mice per group; t = 3.598, df = 30, P = 0.0011. (**) P < 0.01, (***) P < 0.001, (****) P < 0.0001.

**References**:

73 Lübke, J., Egger, V., Sakmann, B. & Feldmeyer, D. Columnar organization of dendrites and axons of single and synaptically coupled excitatory spiny neurons in layer 4 of the rat barrel cortex. *Journal of Neuroscience* **20**, 5300-5311 (2000).

74. Bright, D. & Smart, T. G. Methods for recording and measuring tonic GABAA receptor-mediated inhibition. *Frontiers in neural circuits* **7**, 193 (2013).

75 Anstötz, M. *et al.* Morphology, input–output relations and synaptic connectivity of Cajal–Retzius cells in layer 1 of the developing neocortex of CXCR4-EGFP mice. *Brain Structure and Function* **219**, 2119-2139 (2014).
